# Searching for Solvents with an Increased Carbon Dioxide Solubility Using Multivariate Statistics

**DOI:** 10.3390/molecules25051156

**Published:** 2020-03-05

**Authors:** Marta Bystrzanowska, Marek Tobiszewski, Francisco Pena-Pereira, Vasil Simeonov

**Affiliations:** 1 Department of Analytical Chemistry, Faculty of Chemistry, Gdańsk University of Technology (GUT), 11/12 G. Narutowicza St., 80-233 Gdańsk, Poland; marbystr@student.pg.edu.pl; 2 Department of Analytical and Food Chemistry, Faculty of Chemistry, University of Vigo, Campus As Lagoas-Marcosende s/n, 36310 Vigo, Spain; fjpena@uvigo.es; 3 Chair of Analytical Chemistry, Faculty of Pharmacy and Chemistry, University of Sofia “St. Kl. Okhridski”, J.Bourchier Blvd. 1,1164 Sofia, Bulgaria; vsimeonov@chem.uni-sofia.bg

**Keywords:** ionic liquids, CO_2_ solubility, multivariate statistics, green solvents, cluster analysis

## Abstract

Ionic liquids (ILs) are used in various fields of chemistry. One of them is CO_2_ capture, a process that is quite well described. The solubility of CO_2_ in ILs can be used as a model to investigate gas absorption processes. The aim is to find the relationships between the solubility of CO_2_ and other variables—physicochemical properties and parameters related to greenness. In this study, 12 variables are used to describe a dataset consisting of 26 ILs and 16 molecular solvents. We used a cluster analysis, a principal component analysis, and a K-means hierarchical clustering to find the patterns in the dataset and the discriminators between the clusters of compounds. The results showed that ILs and molecular solvents form two well-separated groups, and the variables were well separated into greenness-related and physicochemical properties. Such patterns suggest that the modeling of greenness properties and of the solubility of CO_2_ on physicochemical properties can be difficult.

## 1. Introduction

The implementation of the green chemistry concept in the design of processes and products is a common practice nowadays. The initial idea was embodied in the form of the 12 principles of green chemistry formulated by Anastas and Warner [1], which mostly refer to organic synthesis. Meanwhile, however, it was noticed that other areas in chemistry may also negatively influence the environment [2]. Bearing in mind the principles of green chemistry, a priority is on the minimization of the use of solvents, the replacement of harmful ones, and recycling, i.e., the so-called “3R” approach—“reduction, replacement, and recycling” [3]. The selection of an appropriate alternative, such as a green solvent (a substance that is not toxic towards human and other organisms, safe, easily biodegradable and recyclable, produced from renewable sources, and with no significant interference with the environment), is a very important and difficult step, in regards to the application of greener methods [4]. It is worth remembering that the finding of a greener option should not only be to provide a replacement, but also additional advantages, such as: improved selectivity, sensitivity, reliability of analysis, or reduction in analysis/reaction time [5].

It is obvious that “the best solvent is no solvent”, even though this is very often practically not feasible. Thus, a number of alternatives to hazardous solvents have been reported in the literature [6,7]. In the last few years, the interest in ionic liquids (ILs)—salts consisting of relatively large asymmetric organic cations and either organic or inorganic anions, with melting points below 100 °C—has increased significantly. That is probably due to the possibility of designing solvents for specific applications, by the combination of appropriate cations and anions. Moreover, ILs are characterized by unique properties, such as a negligible vapor pressure, a high chemical and thermal stability, a low flammability, a wide range of liquidus states, a high ionic conductivity, a large electrochemical window, and an excellent solvation ability of a variety of compounds [8,9]. However, there are also numerous scientific articles in which authors challenged the green nature of ILs, especially based on their poor biodegradability, biocompatibility, and sustainable lifecycle, including their methods of preparation and degradation after use, as well as their impacts on the ecosystem, along with their toxicity [10,11,12,13,14,15,16,17]. Another problem is the lack of data for the characterization of ILs, which makes the evaluation of their greenness difficult.

The knowledge of a given solvent’s properties allows us to find its appropriate application, and to further designate the nuisance and environmental fate of the substance. Sometimes, it may be impossible to easily assess the greenness of a compound, because of insufficient data, and such is the case for ILs. Therefore, some prediction and computational methods to fill the gaps are applied in order to characterize the substances more comprehensively. The easiest manner is chemical predictive modeling that is based on an observation of some patterns in data, which has become a guide for designing future compounds and their properties. For instance, imidazolium salts are nondegradable when alkyl-substituted in positions 1 and 3 [11]. Examples of more advanced and computational methods may be the quantitative structure–activity relationship (QSAR) and estimation programs interface suite (EPI Suite) modeling approaches, which are mostly used for the prediction of chemical properties. QSAR models are used to predict the physicochemical, biological, and environmental fate properties of compounds by reference to knowledge of their chemical structure. The concept involves establishing quantitative relationships between descriptors (representing chemical structure) and the target property capable of predicting the activities of novel compounds [18]. The EPI Suite model is dedicated to the estimation of the physical/chemical and environmental fate properties, such as the water solubility, octanol–water partition coefficient, Henry’s law constant, melting point, boiling point, and aquatic toxicity, based only on the chemical structure as an input (depending on the chosen estimation model program) [19].

In the last few years, the concentration of greenhouse gases as water vapor, carbon dioxide, methane, nitrous oxide, and ozone in the atmosphere has been increasing. A noteworthy compound is CO_2_, which is a significant factor in the global warming and climate change processes [20]. Naturally, CO_2_ in the atmosphere constitutes a part of the Earth’s carbon cycle, which may be absorbed by the oceans, which constitute natural sinks for CO_2_ [21]. The main sources of the CO_2_ emission are the combustion of fossil fuels for transportation, the production of electricity, and some industrial processes, such as cement manufacturing, the processing of natural gas, the iron smelters, and the production of ammonia [22]. A significant amount of anthropogenic CO_2_ is emitted from the flue gases in power plants [23]. Therefore, removing the CO_2_ from flue gases is necessary in order to decrease the carbon level in the Earth’s atmosphere. The well-known solution for CO_2_ mitigation is called CO_2_ capture and storage (CCS), which consists in the capture, transport/utilization, and finally storage/deposit of carbon [24]. Therefore, alternative solvents for CO_2_ capture that could fulfill the principles of green chemistry have been profusely pursued [25]. Solvents for CO_2_ capture should ideally be characterized by a high working capacity, a low regeneration temperature, a viscosity parameter, and a heat of absorption and vaporization [26]. ILs have many of the above properties [27,28,29] and could, therefore, be considered to be very promising solvents for CO_2_ capture. However, there is an issue with their high viscosity, which could be adjusted by an appropriate selection of anions and cations, even though this can also be an advantage, based on the vast number of possible combinations for specific applications [25]. The limitations in the usage of ILs are their potential toxicity and low biodegradability, combined with relatively high costs, which could hinder their industrial-scale application [22]. Since the absorption of CO_2_ by ILs is a well-investigated problem, CO_2_ can be used as a model compound to select ILs as absorbents for gaseous compounds.

The aim of the present study was to apply multivariate statistics to find the patterns among variables and among objects (ILs and molecular solvents), and to discover discriminators for each group. The aim was to find the relationships between the solubility of CO_2_ and the physicochemical parameters of molecular solvents and ILs.

## 2. Results and Discussion

### 2.1. Molecular Solvents and Ionic Liquids

**Figure 1:**
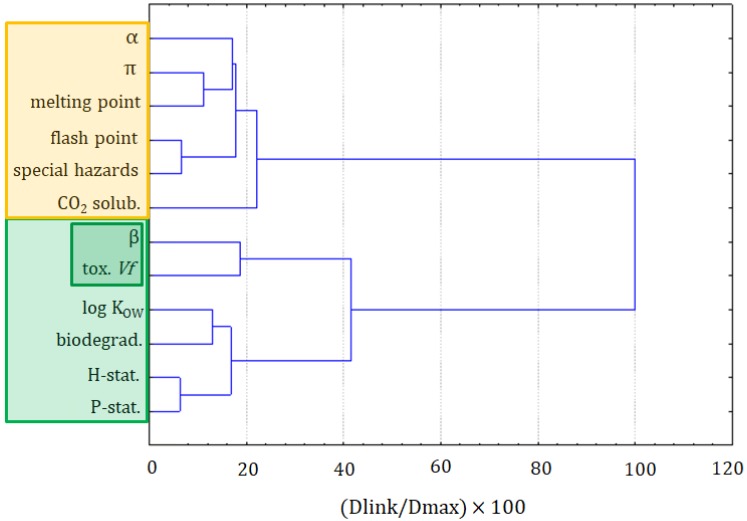
The hierarchical dendrogram for all 12 variables (all ionic liquids (ILs) and molecular solvents are considered).

The first step in this assessment was the clustering of variables and objects with the CA. The clustering of variables is presented in Figure 1. Two major clusters are formed at the distance of two-thirds of D_max_. The first cluster was formed by the following variables: precaution statements, hazard statements, biodegradation, log K_OW_, toxicity towards *Vibrio fischeri*, and the β solvatochromic parameter. The latter two variables could be considered as a separate subcluster. The closest link is between hazards and precautionary statements, taken from MSDSs. It should be seen as indicating that the more serious hazards need more advanced precautionary measures to handle chemicals. The second clear link is between the biodegradability and the log K_OW_, which is explained by the fact that compounds that are more bioaccumulative hardly undergo a biodegradation. The two variables that are loosely connected with others in this cluster are the toxicity towards *Vibrio fischeri* and the β solvatochromic parameter.

The second main cluster is formed by the α and the π solvatochromic parameters, the melting point, flash point, special hazards, and solubility of CO_2_. The closest link is between the flash point and special hazards variables. It can be read as: the compounds that can ignite at a lower temperature also generate more hazardous substances during combustion. Another dependence can be seen in the α and the π solvatochromic parameters and the melting point variables, which is explained by the melting point’s dependence on the polarity and acidity of compounds. The solubility of CO_2_ is only loosely related to this cluster, suggesting that it cannot be easily calculated or attributed to other variables.

The formation of these two clusters reflects the significance of factors related to the toxicity or, more generally, to greenness properties (cluster 1), and to the physicochemical properties of solvents (cluster 2).

**Figure 2:**
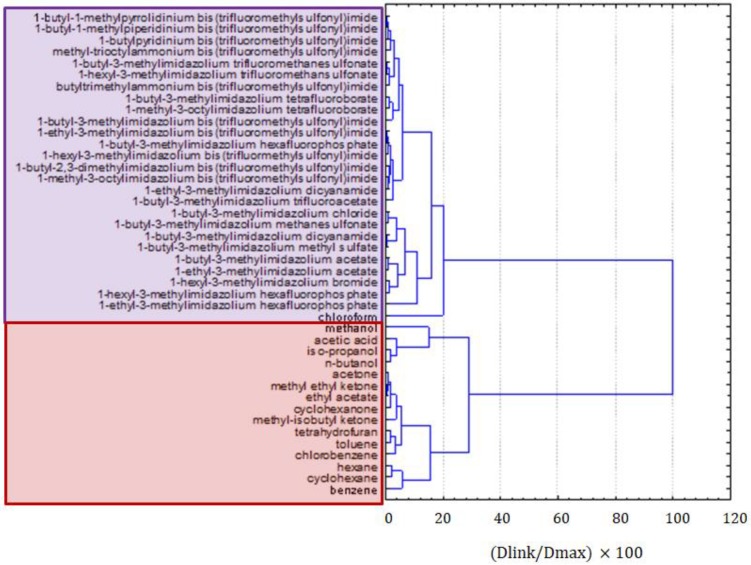
The hierarchical dendrogram for all 42 organic compounds characterized by 12 variables.

Figure 2 presents the clustering of all molecular solvents and ILs together. Two very distinct clusters were formed; the red one was formed by organic solvents, and the purple one consisted of ILs and chloroform. Moreover, a clear grouping of solvents within the red cluster could be observed, according to their molecular structure. The first group was formed by alcohols and organic acids, i.e., solvents of very high polarity, the second group, by ketones and esters, and the third and fourth groups, by aliphatic and aromatic hydrocarbons, i.e., compounds of low polarity. This clear separation of solvents could be considered as a validation that ILs were also well separated in groups of similarity within clusters. There were several groups of ILs consisting of compounds of similar cations and anions, but clear patterns were not easily recognizable. Imidazolium derivatives with bis(trifluoromethylsulfonyl)imide anions were grouped together but pyrrolidinium, piperidinium, and pyridinium salts with the same anions were grouped separately. It seemed that the principal factor by which ILs were clustered is the type of anion, while the type of cation and the length of alkyl substituents played a minor role. More information on the patterns in the cluster of ILs could be obtained by the exclusion of molecular solvents and by grouping ILs separately. Interestingly, chloroform was an outlier in the cluster of ILs, but it was still considered as being more similar to molten salts than to molecular solvents.

**Figure 3:**
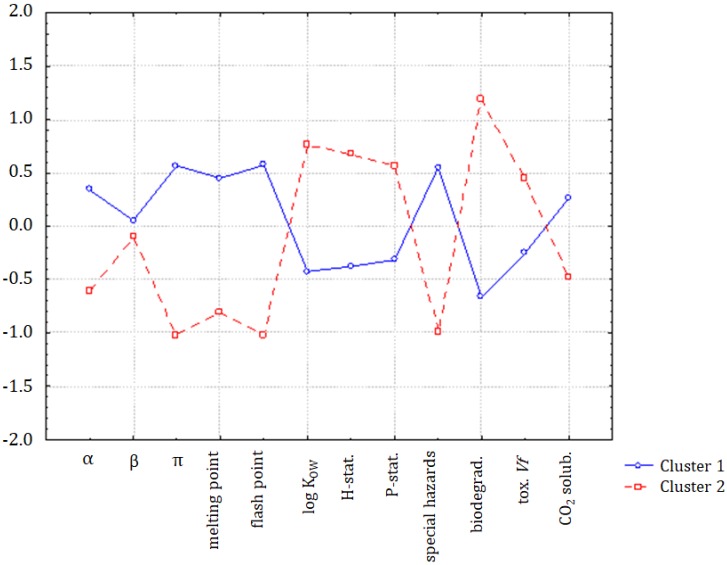
The average values for all variables for each of the identified clusters.

The hypothesis that the group of organic compounds is separated into two distinct classes was confirmed by the application of K-means non-hierarchical clustering. In Figure 3, the discriminating variables for clusters 1 (ILs and chloroform) and 2 (molecular solvents, except chloroform) are indicated. Compounds forming cluster 1 were characterized by higher values of α, π, melting point, flash point, special hazard, and CO_2_ (standardized input data), and the solvents in cluster 2 were characterized by higher values of log K_OW_, hazards and precautionary statements, biodegradability, and toxicity towards *Vibrio fischeri*. Only the parameter β was almost equal for both classes.

The principal component analysis was also performed on the dataset. Three latent factors explained almost 70% of the total variance of the system. The PCA results indicate that the relationship between the variables is very similar to that obtained with the clustering methods. Significant links were found between the variables of precautionary statements, hazard statements, and log K_OW_, then the variables of solubility of CO_2_, melting point, α, and π, and then the toxicity towards *Vibrio fischeri* and special hazards variables.

Since the separation between both classes of organic compounds was significant, it was interesting to separately consider the group of ILs and the group of solvents. This provided a better view on the clustering within the two main clusters.

### 2.2. Ionic Liquids

**Figure 4:**
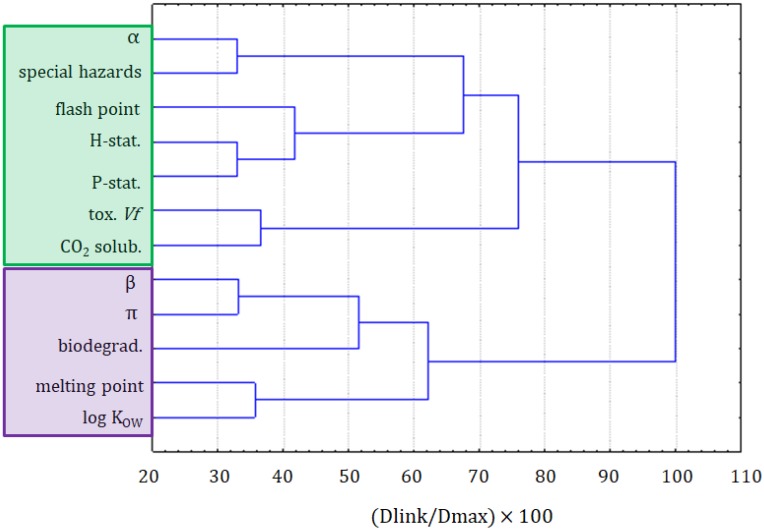
The hierarchical dendrogram for 12 variables. All 26 ILs, as objects, are included.

To investigate the datasets in more detail, the dataset part containing only the information on ILs underwent a cluster analysis. The results of the variable grouping are shown in Figure 4. The linkage of the variables in this case was different, compared with the clustering of all compounds. Two main clusters were formed. The purple one consisted of the β and π solvatochromic parameters, biodegradability, melting point, and log K_OW_. All these variables were connected to the physicochemical properties of ILs. Only the biodegradability could not be considered as a physicochemical variable, but it was related to the physicochemical properties of the compounds. The situation in the green cluster was not as clear. Similarly, as for the clustering of all compounds, it can be seen as hazard and precautionary statements grouped together. Again, this green cluster is more related to the safety of the application of ILs. A separate clustering of variables being the physicochemical properties and variables describing the greenness is a strong implication that the modeling of greenness parameters, on the basis of physicochemical properties, can be difficult. The solubility of CO_2_ is clustered with the toxicity towards *Vibrio fischeri*, the variable that is not convenient to apply for the prediction of the solubility of CO_2_.

**Figure 5:**
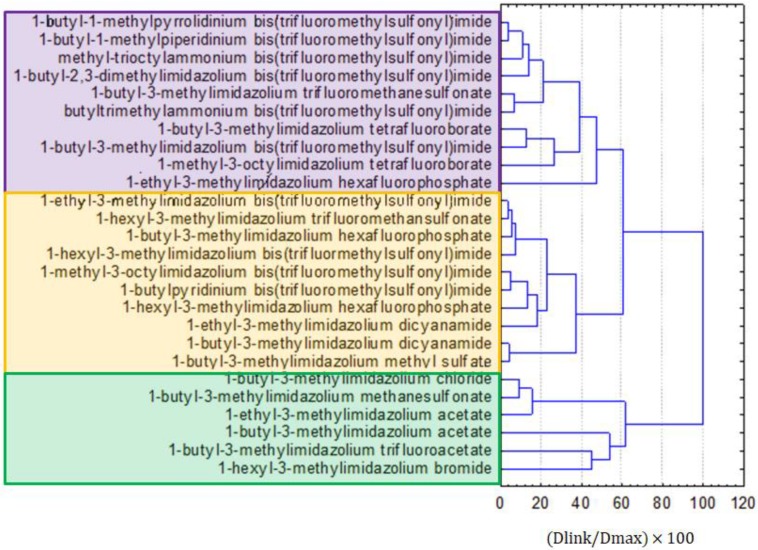
The hierarchical dendrogram for the linkage of 26 ILs.

Three groups of similarity were formed on the dendrogram for the linkage of ILs, as presented in Figure 5. Similarly to the results for the clustering of all compounds, bis(trifluoromethylsulfonyl)imide anion-containing ILs were split into two clusters: purple and yellow, depending on the included type of cation. The green cluster was formed by imidazolium ILs, with relatively small anions. The yellow cluster was mainly formed by imidazolium ILs with larger anions than the compounds from the green cluster. The purple cluster contained ILs with bis(trifluoromethylsulfonyl)imide anions, but other than imidazolium cations and other ILs.

**Figure 6:**
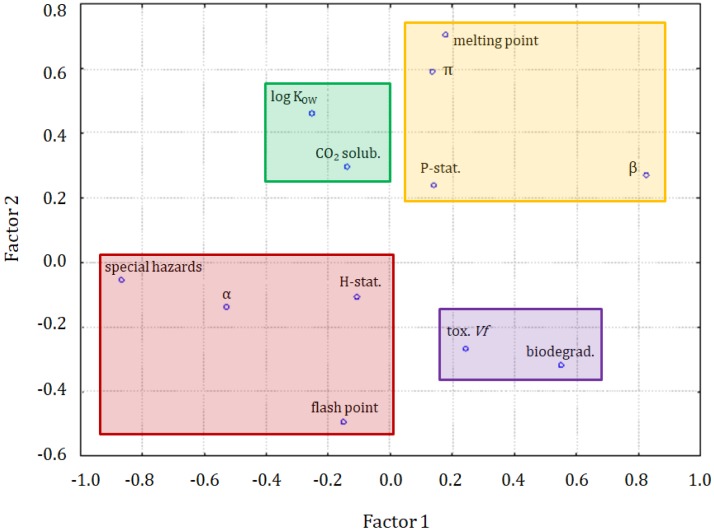
The plot for 3 latent factors.

From the hierarchical clustering and factor analysis, it is evident that a close relationship could be expected between CO_2_ (as a dependent variable) and α, a special hazard arising from the substance or mixture/hazardous decomposition products, the flash point, hazard statements, precautionary statements, and toxicity towards special hazards arising from the substance or mixture/hazardous decomposition products of *Vibrio fischeri* (cluster 1 in the dendrogram in Figure 5), as independent variables or, more specifically, between the solubility of CO_2_ and the log K_OW_ (Figure 6).

**Figure 7:**
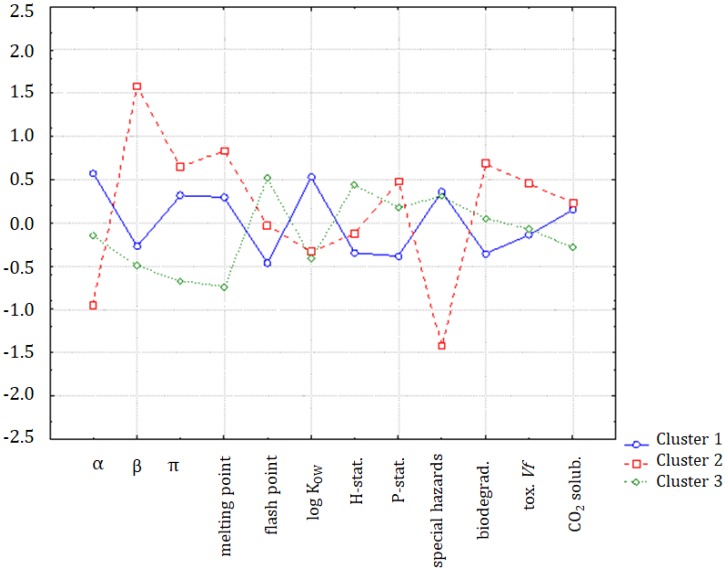
The plot of means for each variable, for each of the identified clusters of ILs.

In Figure 6, the solubility of CO_2_ is in a specific position indicating a close correlation to log K_OW_, but also a weaker correlation with the α solvatochromic parameter, flash point, hazard statements, and special hazards arising from the substance or mixture/hazardous decomposition products. It can be seen, in Figure 7, that the major differences between clusters were due to the variability of the β parameter, melting point, log K_OW_ to some extent, special hazards, and biodegradability.

### 2.3. Molecular Solvents

**Figure 8:**
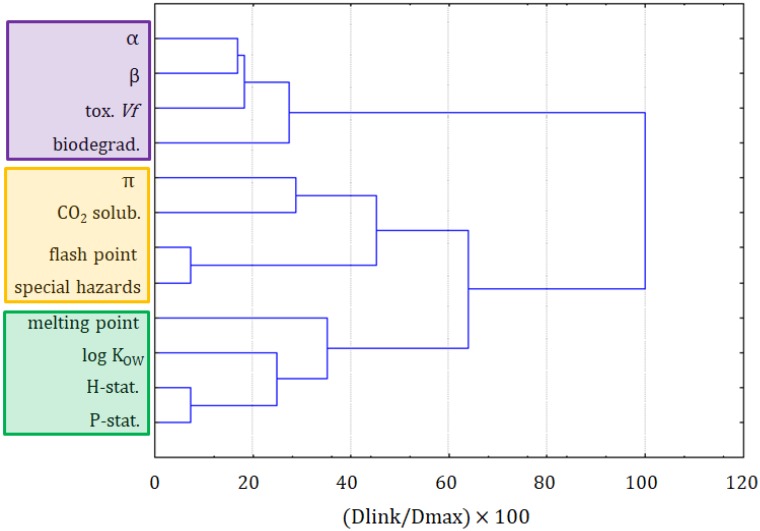
The hierarchical dendrogram for the clustering of 12 variables. Only the molecular solvents are included as objects.

**Figure 9:**
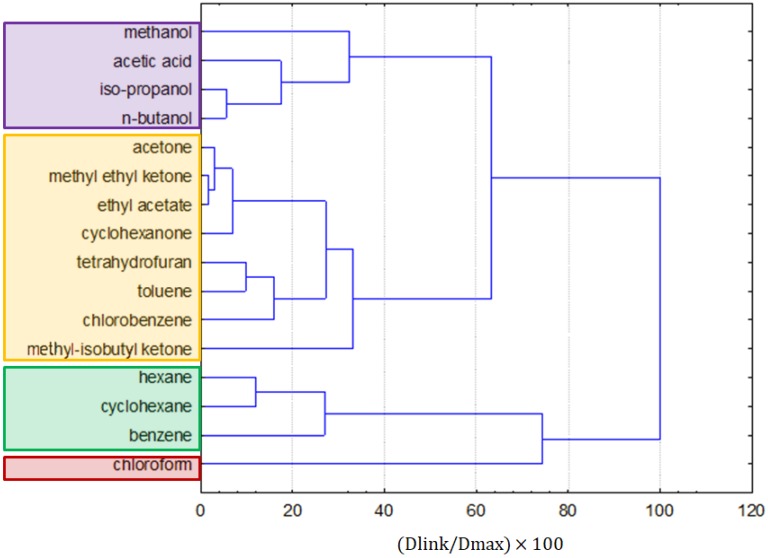
The hierarchical dendrogram for the linkage of 16 solvents.

If only the molecular solvents were involved in the multivariate statistical analysis, the following results could be interpreted according to Figure 8 (the clustering of variables) and Figure 9 (the clustering of objects). The clustering of variables differed from the clustering of ILs, but a few patterns were similar. In the green cluster, again, the hazard and precautionary statements were closely linked, together with the melting point and the log K_OW_. Again, the pattern of the special hazards and flash point linked together could be observed in the yellow cluster. They were clustered with π and the solubility of CO_2_. The purple cluster consisted of the α and the β solvatochromic parameters, and of the *Vibrio fischeri* toxicity, with the biodegradability.

In the case of the solvents, the solubility of CO_2_ was related to the π solvatochromic parameter, which implies that the solubility of CO_2_ can be modeled with this solvatochromic parameter as one of the input variables.

The clustering of solvents (Figure 9) generally kept similar groups of clustering of molecular solvents and ILs together. The purple cluster was formed by polar alcohols and acetic acid, and the yellow one was formed by less-polar ketones, ethyl acetate, toluene, chlorobenzene, and tetrahydrofuran. The green cluster was formed by the non-polar solvents hexane, cyclohexane, and benzene. An outlier is chloroform, which was found to be an outlying object in previous clustering (being more similar to ILs), and it was arbitrarily included in this cluster analysis, as it is a molecular solvent.

**Figure 10:**
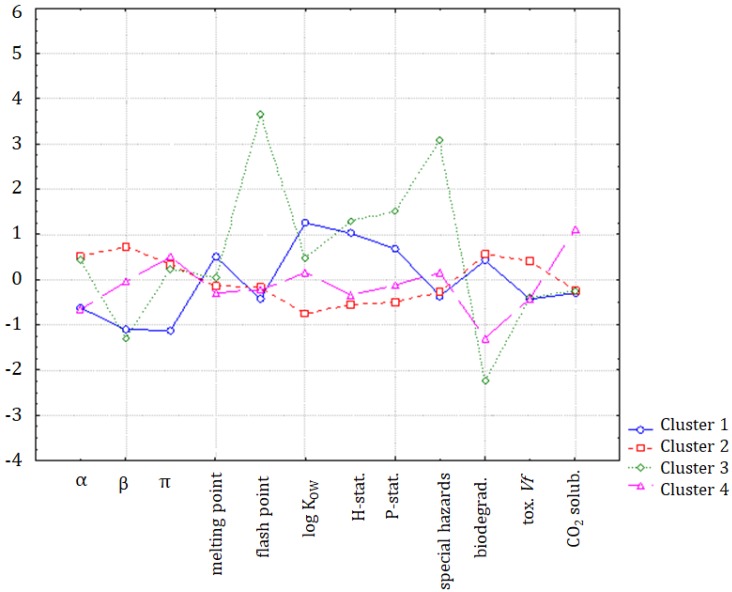
The average values (standardized) for each variable, and for each identified cluster.

If the K-means non-hierarchical clustering is applied following the hypothesis of the formation of four patterns of similarity, the members of the first cluster are: hexane, benzene, toluene, and cyclohexane. The second cluster is formed by methanol, acetone, acetic acid, methyl ethyl ketone, iso-propanol, n-butanol, ethyl acetate, and cyclohexanone. The third cluster is formed by chloroform, which may also be considered to be an outlying object, and the fourth cluster is formed by tetrahydrofuran, chlorobenzene, and methyl-isobutyl ketone. The K-means non-hierarchical clustering yielded very similar results to the cluster analysis. To investigate the main differences between clusters, a discriminant analysis was applied. The discriminant variables for each cluster could be interpreted from Figure 10. In general, clusters 1, 2, and 4 were quite similar, and only the chloroform (outlier) indicated significant differences due to the flash point, special hazards, and biodegradability parameters.

## 3. Materials and Methods

### 3.1. Dataset

The initial dataset consisted of 42 compounds; 26 of them were ILs and 16 were molecular solvents, which were characterized by 12 variables. More ILs were initially considered in the study, but their number was limited by the availability of data, mainly on the solubility of CO_2_. Molecular organic solvents were selected in such a way that different classes of chemical compounds and solvents of various hazards were covered. The variables that described these solvents were the α, β, and π solvatochromic parameters, and the melting point, flash point, log K_OW_, biodegradability, toxicity towards *Vibrio fischeri*, and solubility of CO_2_ in a solvent. Three additional parameters were taken from the material safety data sheets (MSDSs)—hazard statements, precautionary statements, and special hazards arising from the substance or mixture/hazardous decomposition products. Since this information was of a descriptive character, it was transformed into numerical values using the approach presented in a previous study [30].

### 3.2. Cluster Analysis

One way to classify solvents is to use a well-known and widely used multivariate statistical approach, namely the cluster analysis (CA) [31]. It is a tool for environmetric, food, and various other product characterization, grouping, or clustering purposes. In order to cluster objects that are described by a set of variables (in this case, mostly the physicochemical parameters of ILs and molecular solvents), one has to calculate their similarity. The first step is the transformation of data, such as the auto-scaling or z-transformation, and range scaling, where real data values are substituted with normalized dimensionless numbers. As a result, the differences in absolute values, expressed in different units, are scaled to the same range. Consequently, the similarity between many objects in a multidimensional space can be calculated. In this study, the Euclidean distance was frequently used as a measure. As a result, the raw data input matrix was transformed into a similarity matrix. Ward’s method was selected as the method for linking the objects of similarity into clusters. The CA results are presented as a tree-like scheme called a dendrogram, which clearly reflects a hierarchical structure of the initial database. In the present study, the clustering was performed with Ward’s mode of linkage and the squared Euclidean calculation of distances. CA calculations were performed using the Statistica 12 software (Stat Soft, Inc., Palo Alto, CA, USA).

The clustering approach described above is a representative of the so-called hierarchical agglomerative cluster analysis (HCA), which is a typical non-supervised pattern recognition method. In our study, the non-hierarchical cluster analysis (the K-means mode) was also used as a supervised pattern recognition method, where the numbers of clusters to be interpreted were preliminarily defined, due to some hypothesis concerning the data structure. Again, the patterns of similarity were revealed for objects of observation or between the variables characterizing the objects. The K-means mode aimed to minimize the internal cluster variances (represented by the squared Euclidean distances between the objects or variables of interest). The algorithm was related to the K-nearest neighbor classifier (KNN classifier) used in machine learning classification problems.

### 3.3. Principal Component Analysis

Another widely used technique for reducing the number of dimensions is the Principal Component Analysis (PCA). The aim of applying this technique is to extract the significant information from a large dataset, thus increasing the clarity and interpretability of the data, without much loss of information. This aim is reached by the creation of new uncorrelated variables that keep the variance in a maximal way. The new variables are orthogonal, which expresses the original information without much loss, and they are named principal components (PCs). The similarity between these elements can be shown in Cartesian coordinates. The original dimensions of the input matrix were reduced to factor loadings and factor scores. Factor loading included the weight of each variable in each new factor. As a consequence, when the value of this weight was high, it meant that the original variable contribution was also significant. PCA calculations were performed using the Statistica 12 software.

## 4. Conclusions

Multivariate statistics allowed us to find the internal patterns in a dataset of solvents, including ILs and molecular solvents. The grouping of variables for all compounds, and for molecular solvents or ILs separately, showed very different patterns. This means that ILs and molecular solvents cannot be directly compared in terms of physicochemical properties or variables related to their greenness.

The solubility of CO_2_ in ILs cannot be easily modeled with physicochemical properties, as, according to the cluster analysis, the closest link is for the toxicity towards *Vibrio fischeri*. In the case of molecular solvents, the closest link is for the π solvatochromic parameter. The PCA results showed that the solubility of CO_2_ in ILs could be related to the log K_OW_, which is a convenient parameter for prediction studies.
